# Effects of an emotional literacy intervention for students identified with bullying behaviour

**DOI:** 10.1080/01443410.2013.785052

**Published:** 2013-05-07

**Authors:** Claire Knowler, Norah Frederickson

**Affiliations:** a Milton Kaynes Council, Educational Psychology Service, Milton Keynes, UK; b UCL, London, UK

**Keywords:** emotional literacy, emotional intelligence, bullying, intervention, primary school

## Abstract

The effectiveness of a 12-week, small group emotional literacy (EL) intervention in reducing bullying behaviour in school was evaluated. Participants were 50 primary school pupils identified through peer nomination as engaging in bullying behaviours. The intervention was implemented in schools already engaged with a universal social and emotional learning initiative, including an anti-bullying component. Within schools, participants were randomly assigned to an intervention or a wait-list comparison group. Response to the intervention was found to be dependent on baseline levels of EL. Only children whose baseline level was low showed a significant reduction in peer-rated bullying behaviour. No effect of the intervention was detected on victimisation or adjustment scores, although positive changes in adjustment were associated with increased EL.

## Introduction

Bullying in schools has become an issue of concern internationally (Nansel, Craig, Overpeck, Saluja, & Ruan, 2004; Smith, Pepler, & Rigby, 2004). There are many definitions of bullying, but substantial consistency among western researchers in identifying bullying as a subset of aggression, which usually involves the following core characteristics: (1) intentional harm, (2) repetition over time and (3) occurring in a relationship where there is an imbalance of power (Nansel & Overpeck, 2003; Olweus, 1999). These features are correspondingly frequently found in government guidance to schools, for example, in the United Kingdom (UK) bullying is defined as ‘behaviour by an individual or group, repeated over time, that intentionally hurts another individual or group either physically or emotionally’ (Department for Education, 2011, p. 4). However, these features have been subject to criticism at a number of levels, and a range of arguments advanced for the adoption of a broader conceptual approach (Carrera, DePalma, & Lameiras, 2011; Ellwood & Davies, 2010; Juvonen & Graham, 2004). Consistent with socio-ecological perspectives on the complex nature of the interactions between the individual, family, peer-group, school, community and culture (Hong & Espelage, 2012; Swearer & Espelage, 2004) the importance of attending to the different meanings constructed and ascribed, in particular by pupils asked to respond to researcher's definitions, has been highlighted (Carrera et al., 2011; Swain, 1998).

There is widespread recognition of the impact of bullying on children who are victims, as damaging to their psychological, social, academic and physical development (Marsh, Parada, Craven, & Finger, 2004; Pellegrini, 2004). However, children who bully others also fare badly. They report elevated levels of depression, unhappiness at school and family conflict (Oliver, Oaks, & Hoover, 1994). They have increased risk of associated emotional and behavioural problems (Salmon & Smith, 1998), and may suffer from depression and from suicidal thoughts and intentions (Kaltiala-Heino, Rimpela, Marttunen, Rimpela, & Rantanen, 1999). Furthermore, children exhibiting bullying behaviours are at elevated risk of engagement in criminal behaviour in later life (Olweus, 1991, 1993).

Despite the substantial evidence that there are negative consequences for children who engage in bullying, few interventions are designed for such children. The view that punishment is the most appropriate approach to children who bully has led to controversy over interventions that seek to adopt a more educative approach (Smith, 2001), such as bringing together all pupils involved in a sequence of bullying events to elicit concern for others and to problem solve more acceptable ways of interacting in the future (Pikas, 2002; Rigby, 2005). Frey et al. (2005) identify a need for studies to consider targeted interventions, rather than purely universal approaches. They question the extent to which schools provide intensive, individually-focused interventions for children who need more direct guidance. While universal supports available to all children are regarded as an essential basis in three-tiered public health prevention and intervention models (Walker et al., 1996), it is also considered important for schools to be able to provide targeted interventions for children at risk. Indeed, response to such intervention is seen as playing a crucial role in the effective selection of children in need of third tier, individualised, intensive intervention (Hawken, Vincent, & Schuman, 2008).

Some of the best known bullying intervention programmes incorporate universal and targeted interventions, for example, both the Olweus (1999) and the Steps to Respect (Committee for Children, 2001) programmes include work with individuals involved in bullying as well as school-wide and classroom components. However, there are programmes where the focus is systemic and individual level intervention is not included, for example the PEACE Pack (Slee, 2001) and the Sheffield Programme (Smith Sharp, Eslea, & Thompson, 2004). Meta analyses of intervention effectiveness have indicated moderate outcomes, reflecting predominately positive changes in knowledge, attitudes and perceptions, although more rarely showing change in bullying behaviours (Merrell, Gueldner, Ross, & Isava, 2008; Ttofi & Farrington, 2011). While Merrell et al. (2008) did not find any evidence of differential effectiveness by type of programme, Ttofi and Farrington (2011) found that programmes containing more elements were more likely to reduce bullying.

The present study reports the evaluation of a pilot implementation of a targeted bullying intervention within the tiered framework described by Walker et al. (1996). It was implemented in schools that were already using the Social and Emotional Aspects of Learning (SEAL) Programme (Department for Education and Skills, 2005) – a programme comprising professional development materials for teachers, curriculum materials for each grade, a whole school component involving policy presentations and posters and home-school activities. The SEAL programme addresses five themes, one of which is ‘say no to bullying’. However, other themes focus more broadly on issues such as managing relationships, motivation and developing effective learning strategies, thus representing a more holistic approach to school improvement of the kind advocated by Galloway and Roland (2004) as preferable to a narrow focus on bullying behaviour. Within this context, the targeted intervention investigated in this study was selected on the basis of an analysis of what is known about children who engage in bullying behaviours that points to deficits in aspects of emotional intelligence.

### Emotional intelligence

In defining emotional intelligence a distinction is drawn in the literature between *ability emotional intelligence* and *trait emotional intelli*gence and constructs that stem from different theoretical positions and are assessed in different ways (Petrides & Furnham, 2000; Warwick & Nettelbeck, 2004). Ability emotional intelligence refers to actual emotion-related abilities and should be measured through maximum performance tests, as are used for the measurement of psychometric intelligence (Mayer, Caruso, & Salovey, 1999). However, there are problems in the identification of correct responses for ability emotional intelligence, reflecting the inherently subjective nature of much emotional experience. More progress has been made, particularly with children, in the assessment of trait emotional intelligence (trait EI), or trait emotional self-efficacy (Williams, Daley, Burnside, & Hammond-Rowley, 2009). Trait EI refers to ‘emotion related self-perceptions and behavioural dispositions relating to the perception, processing, and utilisation of emotion-laden information’ (Mavroveli, Petrides, Sangareau, & Furnham, 2009, p. 259). Trait EI is assessed through self-report measures of typical performance on dimensions such as emotion perception (self and others), emotion regulation, relationship skills and empathy (Petrides & Furnham, 2000).

The relevance of trait EI to behavioural issues in school has been suggested by a number of studies. Petrides, Sangareau, Furnham, and Frederickson (2006) investigated the relationship between trait EI and social behaviour in primary school as rated by peers and teachers. Pupils with high trait EI scores received more peer nominations for co-operation and leadership and fewer nominations for disruption, aggression, and dependence. Teachers rated high trait EI pupils higher on pro social descriptors and lower on antisocial descriptors, than pupils who had low trait EI scores. In 10 to 11-year-old primary school children, trait EI scores have also been found to correlate with self-reported measures of adjustment: positively with self-concept, and negatively with depression, anxiety, anger and disruptive behaviour (Williams et al., 2009). Qualter, Whiteley, Hutchinson and Pope (2007) found that pupils who had low trait EI scores experienced greater adjustment difficulties in the transition between primary and secondary school. Secondary school pupils with high trait EI scores were found to be less likely than those with low scores to have unauthorised absences from school or to have had periods of exclusion from school due to rule violations (Petrides, Frederickson, & Furnham, 2004).

### Children who engage in bullying behaviour

Consistent associations are reported between bullying and other behaviour problems, for example conduct problems (Viding, Simmonds, Petrides, & Frederickson, 2009), and externalising (Andreou, 2001) or arousal seeking behaviour (Woods & White, 2005). In addition, bullying is frequently associated with peer problems and low levels of prosocial behaviours (Arseneault et al., 2006; Wolke, Woods, Bloomfield, & Karstadt, 2000). Wolke et al. (2000) reported similar patterns of associations for both direct and indirect bullying in their 6–9 year old sample and, like Craig (1998), found that boys of this age were more frequent perpetrators of both types of bullying. Across childhood and adolescence higher 2levels of engagement by males in direct aggression is supported, but stereotypical attribution of higher rates of indirect aggression to females challenged, by meta analytic findings (Archer, 2004; Card, Stucky, Sawalani, & Little, 2008), and the need to consider the relationship between gender and bullying in developmental and cultural context highlighted (Carrera et al., 2011; Felix & Grief-Green, 2010). Archer (2004) reported that girls were only found to be more involved in relational aggression in samples above 11 years, where bullying was assessed using peer ratings. Boys were more likely to engage in physical bullying than girls cross all ages.

Gender considerations are also relevant to the relationship between bullying and socially skilled behaviours and cognitions (Garandeau, Wilson, & Rodkin, 2010), where there are contradictory findings. Some have argued that children who bully are socially unskilled (Crick & Dodge, 1999), but others report that they tend to fall into two groups: *socially skilled*, and *socially unskilled* (Kaukiainen et al., 2002). However, socially skilled or unskilled behaviours represent the outcome of interactions between individual competencies and the social demands of situations (Beauchamp & Anderson, 2010). Taking account of situational factors, the two groups identified by Kaukiainen et al. (2002) appear to map onto the distinction drawn by Olweus, (1993) between ‘bully-victims’ defined as children who bully others in some situations but are themselves bullied in others, and ‘pure bullies’ who bully others in some situations but are not themselves. It has been suggested that bully-victims may have a social skills deficiency that leads to engagement in highly emotional reactive aggression in response to real or perceived threats. Pure bullies, on the other hand, may be more cold and calculating, possess good social cognition, and engage in proactive aggression driven by anticipated rewards and controlled by reinforcement contingencies (Sutton, Smith, & Swettenham, 1999). However, this individually-focused analysis may be overly simplistic. Aspects of the context, such as the role played by bystanders, can be crucial in creating a threatening or supportive environment (Cowie, Hutson, Oztug, & Myers, 2008) and in delivering consequences contingent on bullying behaviour (Cowie, 2009; Nickerson, Mele, & Princiotta, 2008).

In addition, so-called pure bullies may also exhibit emotion-related deficits. Gini (2006) found that children who bully did not have difficulties with a social-cognition task but were more ready to show moral disengagement mechanisms. Warden and Mackinnon (2003) found that bullies were less aware than pro-social children of the possible negative consequences of strategies they chose in problematic social situations. Such findings suggest that what bullies may lack, and what may differentiate them from pro-social children, is the ability to appreciate the emotional consequences of their behaviours on others’ feelings, and to share in and empathise with the feelings of others (Arsenio & Lemerise, 2001). In line with this perspective, a negative relationship has been found between bullying behaviour and empathy (Jolliffe & Farrington, 2006). It seems therefore that for different groups of children who engage in bullying behaviours there is evidence of deficits on dimensions of emotional intelligence such as emotion perception, emotion regulation, and empathy (Petrides & Furnham, 2000; Humphrey, Curran, Morris, Farrell, & Woods, 2007). However, intervention programmes designed to improve EI have not yet been applied to the problem of bullying.

### Emotional literacy interventions

Analogous to the well established use of *thinking skills* programmes to teach the reasoning and other cognitive abilities assessed by traditional intelligence tests (Adams, 1989; Dewey & Bento, 2009; Romney & Samuels, 2001), a number of *emotional literacy* (EL) programmes have been developed to teach knowledge and skills from the sampling domain of EI, such as those required to ‘recognise, understand, handle and appropriately express emotions’ (Sharp, 2001). However the extent to which many of these EL programmes are rooted in emotional intelligence theory has been questioned by Zeidner, Roberts, and Matthews (2002) who found that few programmes were specifically designed to change EI. In addition, very few evaluation studies have actually used EI measures, instead improvements in EI have been inferred from outcomes predicted to be influenced by improved EI, such as reductions in aggressive behaviour (Humphrey et al., 2007). It would seem important therefore that an evaluation of an EL intervention should both assess and target key components of the conceptual EI framework underpinning the programme.

### The current study

The current study aimed to evaluate the effects of a targeted EL intervention on bullying behaviour, trait EI and behavioural adjustment. Given that some children who engage in bullying behaviours are themselves bullied, it was also considered important to investigate the effects of the intervention on victimisation. While it has been argued that different types of children who bully may benefit from an EL intervention, this may well depend on their pre-existing level of EL. Qualter et al. (2007) found that an EL intervention designed to counter negative effectives of transition from primary to secondary school was effective for pupils with low, but not high, baseline scores on trait EI. It was therefore hypothesised that an EL intervention based on the broad sampling domain of trait EI would produce a reduction in bullying behaviour and victimisation, but an increase in trait EI and adjustment, and that the intervention effect would be greater for those children whose EL scores were initially low. It was also hypothesised that reductions in bullying and victimisation and improvements in adjustment would be associated with increases in emotional intelligence and EL, particularly among children who had received the EL intervention. As bullying interventions have been reported to be more effective in primary schools (Pepler, Smith, & Rigby, 2004; Smith, Ananiadou, & Cowie, 2003), it was decided to carry out this pilot study with pupils of primary school age.

In summary, the following hypotheses were investigated.

(1) *Bullying behaviours and victimisation will*: (a) decrease for participants receiving the EL intervention, relative to those in a wait-list comparison group and (b) decrease more for intervention group participants initially scoring low on EL, than for those initially scoring high on EL.
(2) *Trait EI will*: (a) increase for participants receiving the EL intervention, relative to those in a wait-list comparison group and (b) increase more for intervention group participants initially scoring low on EL, than for those initially scoring high on EL.
(3) *Indices of adjustment will*: (a) improve for participants receiving the EL intervention, relative to those in a wait-list comparison group and (b) improve more for intervention group participants initially scoring low on EL, than for those initially scoring high on EL.
(4) Reductions in bullying and victimisation, and improvements in adjustment, will be associated with increases in trait EI and EL scores, particularly in the intervention group.

## Method

### Participants

Fifty children aged 8–9 years were identified by a peer nomination measure of engagement in bullying behaviour. The threshold for selection of the intervention was set at identification by at least 10% of classmates, so children would be independently identified as presenting significant bullying behaviour by at least three of their classmates. On account of constraints on intervention group size, a maximum of seven children from each of the eight classes involved were recruited for this study. The children were randomly assigned to either an intervention or wait-list comparison group. Three children were withdrawn as parental consent was not available for participation in the intervention, and two were withdrawn during the intervention period because they moved out of the area. The 10% threshold level of peer nominations was substantially exceeded by most participants in each condition, (intervention group: *M* = 33.1, SD = 18.0, comparison group: *M* = 39.7, SD = 18.9).

The intervention and wait-list comparison groups were broadly equivalent across a range of demographic variables: gender, majority or minority ethnic group membership and attainment in English and mathematics. There were 22 children (18 boys and 4 girls) in the intervention condition and 23 (21 boys and 2 girls) in the wait-list comparison condition. The gender split reflects previous research on bullying in which boys are more identifiable as bullies as they tend to engage in direct bullying involving physical or verbal attacks (Olweus, 1993). The intervention group comprised 68% White British children, 14% Black Caribbean and African, 9% Indian and 9% other minority ethnic and mixed heritage groups. The comparison group comprised 65% White British children, 23% Black Caribbean and African, 4% Indian and 9% other minority ethnic and mixed heritage groups. Information from school records indicated a representative spread of attainment in English and mathematics for pupils of this age within each group.

Eligibility for free school meals was collected as an index of socioeconomic status. In the wait-list comparison group 13% (*n* = 3) of pupils were found to be eligible, a figure closely comparable with the percentage reported for primary schools nationally (14.5%, Hansard. 2007). However, none of the pupils in the intervention group were recorded by their schools as eligible for free school meals. None of the children in the study had legally ascertained special educational needs for which school district level provision was mandated. However, across both groups a number of children had recorded special educational needs at a lower level, necessitating additional action by their schools, sometimes with consultation or support form district special educational services. This was the case for 36% of the children allocated to the intervention group and 31% of those allocated to the comparison group. School-based interventions previously provided for these children had included small group circle time, anger management, and individual mentoring.

### Procedure

Approval for the project was obtained from the university ethics committee, and from the school district in which the study took place, a suburban area of a large English town. Four primary schools were invited to take part in the study, and all agreed to do so. These schools were known by the first author to be actively engaged with a universal social-emotional learning programme which included an anti-bullying component. Each had two classes in each year group, with approximately 30 pupils per class. Parents of all children in these classes received a letter from their child's school informing them that two whole class assessment sessions would take place, advising them that the information would be made available to school staff for monitoring of bullying behaviour and giving them the opportunity to withdraw their child or to contact the first author for further information. No parent sought further information or opted to withdraw their child.

Whole class measures (Guess Who bullying and victimisation) were carried out by the first author in the classroom in the presence of the class teacher. The purpose of the activities was explained to the children in terms of helping schools promote positive relations and reduce bullying. The voluntary nature of their participation was explained to the children and assurances about anonymity provided in age-appropriate language. No child declined to participate. The importance of confidentiality was stressed and, following administration of the Guess Who measures, the first author and class teacher delivered a personal social and health education lesson focused on strategies for dealing with bullying.

Using the Guess Who bullying measure children eligible for the intervention were identified as described in the participants section. Within each school, participants were randomly assigned by coin toss to one of two groups: an intervention group, and a wait-list comparison group. Information about the intervention and its delivery in two phases was given to parents of these children. Active parental consent was required for the participation of each child in the programme and access to their school records. Three of the 50 parents from whom consent was sought declined to permit their child's participation.

The first author met with the children for whom parental consent had been obtained to explain the intervention and the study and to seek their consent for participation. No child declined to participate in the intervention. All children involved in the intervention and wait-list comparison groups also agreed to complete the other study measures and these were administered by the first author pre-intervention and post-intervention. Following the conclusion of the twelve-week intervention the first author again visited each classroom to re-administer the Guess Who bullying and victimisation measures.

### Intervention

The targeted intervention described below was delivered in this study in the context of the active engagement of each of the four schools with the *SEAL* programme (Department for Education and Skills, 2005), a national social and emotional learning initiative in the UK. Primarily, a universal preventative approach, the SEAL programme also provides some differentiated resources for small group work with children who need extra support. The programme addresses seven themes, including *saying no to bullying*, so the intervention described in this study was implemented against a background of whole school and class-based work on social and emotional learning and bullying. The importance of such congruence between novel targeted interventions and existing practice in schools has consistently been highlighted (Elias, Zins, Gaczyk, & Weissberg, 2003; Weare & Gray, 2003).

The targeted intervention was a programme that involved the explicit teaching in small groups of EL skills taken from the *Emotional Literacy Assessment and Intervention Ages 7–11* Pack (Faupel, 2003). In order to facilitate the implementation of the programme by trained school staff, a scheme of work was written by (and is available from) the first author. The scheme focused on four sections of the above intervention: (1) Developing self-awareness, (2) Learning about self-regulation, (3) Enhancing empathy and (4) Improving social skills. There were three sessions on each theme, so the programme of weekly sessions required 12weeks to complete. For example, in the theme on developing self-awareness, the three sessions focused on recognising strengths, recognising and identifying feelings, and emotions and behaviour, respectively. Each session was delivered in school time and lasted 45–60 min. The scheme of work, although largely based on the EL Intervention (Faupel, 2003), had a particular focus on behavioural and cognitive-behavioural elements: for example, the children were set weekly tasks to complete which involved noting linked behaviours, thoughts and feelings. There were two groups of five and two groups of six children and the main emphasis of the group sessions was on discussion, role-play and practical activities.

The intervention was delivered in school time by teaching aids, para-professionals who were permanent members of school staff. Their role in the schools involved supporting children's engagement and learning in the classroom and implementing small group interventions. Children in these schools were very used to participating teaching aid led small group work linked either to the national literacy and numeracy programmes or, as in this case, to the SEAL programme, so no particular explanation was needed for these group sessions. The children in each class who were not involved with the intervention remained with their class teachers, following their normal classroom curriculum. To promote integrity of implementation across schools, a two-hour training session was provided by the first author and attended by all school staff responsible for implementing the programme. The initial EL session was observed by the first author in each school and telephone consultations and drop-in support were provided throughout the study

Fidelity of implementation was assessed in two ways. Firstly, through discussion of a record completed by the school staff, to show how closely each session adhered to the lesson plan from the scheme of work. Secondly, through periodic observation by the first author, and scrutiny of pupil worksheets completed during the sessions. The first session was observed by the first author in all cases to establish that the staff could be relied on to implement the intervention. Feedback was also given on the sessions observed. A fidelity questionnaire was completed for each session in discussion with school staff, drawing on the records they had completed and on the worksheets completed by the pupils. A rating was given on a 5-point scale (from 1: *very different from the plan* to 5: *exactly the same as the plan*) to indicate how closely each session had been implemented according to the lesson plan from the scheme of work. A median fidelity of implementation score was calculated across all sessions, giving a score ranging from 1, which was considered poor, through satisfactory, good, and very good, to 5, which was considered excellent. Median session ratings were *good* for two of the four schools and *very good* for the other two. No session was rated lower than satisfactory.

### Measures

#### Bullying and victimisation

Investigations into bullying behaviour typically use either self-report or peer nomination methods (Cornell, Sheras, & Cole, 2006). Studies comparing these methods have found that children who engage in bullying typically report lower levels than peers who rate them (Pakaslahti & Keltikangas-Jarvinen, 2000). To guard against such under-reporting the present study utilised a peer nomination method. The Guess Who peer assessment procedure used by Coie and Dodge (1988) was adapted, following Parkhurst and Asher (1992), to allow unrestricted nominations and use proportion scores. The bullying and victimisation items written for the Guess Who procedure by Nabuzoka and Smith (1993) were used. Children were asked to identify anyone in their class who fitted the following behavioural descriptors:

*Bully*: ‘This person is a bully and often picks on other people or hits them, or teases them, or does other nasty things to them for no good reason’.

*Victim*: ‘Someone who often gets picked on, or hit, or teased, or has nasty things done to them by other children for no good reason’.

The score for each child was the proportion of classroom peers who nominated them for each descriptor.

*Emotional Literacy Assessment-Pupil Form* (*ELA-PF*) (Faupel, 2003) is a self-report measure of EL for children aged 7–11 years, standardised in the UK. The pupil form contains 25 items mapped on to the components of EL as defined by Goleman (1996): self-awareness, self-regulation, motivation, empathy and social skills. This assessment is linked to the intervention implemented in this study and both focus on the same aspects of EL. A 4-point rating scale is used to indicate how true each item is for the pupil: *very true, somewhat true, not really true* or *not at all true.* In the current study, a Cronbach's alpha coefficient of .63 was obtained. High and low EL groups were identified by splitting the scores at the median.

*Trait Emotional Intelligence Questionnaire-Child Form (TEIQue-CF)* (Mavroveli, Petrides, Shove, & Whitehead, 2008) is a multidimensional questionnaire designed for completion by children aged 8–12 years. The questionnaire contains 75 short statements (e.g. ‘If I'm sad, I try to put on a happy face’), responded to on a 5-point Likert scale from *completely disagree* to *completely agree.* Items were designed to cover nine facets of trait EI: adaptability, affective disposition, emotion expression, emotion perception, emotion regulation, low impulsivity, peer relations, self-esteem and self-motivation. This measure therefore has a broader sampling domain of EI than the aspects covered by the EL measure (ELA-PF). In the current study, the Cronbach's alpha coefficient was .79.

#### Adjustment

*The Strengths and Difficulties Questionnaire Self-Report Version* (*SDQ*) (Goodman, 1997). The SDQ is a measure of adjustment and psychopathology for children and young people that is widely used in UK educational and clinical settings. The questionnaire contains 25 items organised into five scales (five items per scale): emotional symptoms, conduct problems, hyperactivity and inattention, peer relationship problems and pro-social behaviour. Items are rated on a three-point scale as *Not true, Somewhat true or Certainly* true. Subscale totals are the sum of the scores for the 5 items (0–10). A *Total Difficulties* score is obtained from the sum of the four subscales: emotional symptoms, conduct problems, hyperactivity and inattention, peer relationship problems. Originally designed for young people aged 11–16, there is evidence of sound inter- informant and test–retest reliability from 7 to 8 years old (Mellor, 2004; Muris, Meesters, Eijkelenboom, & Vincken, 2004). In the current study, the Cronbach's alpha coefficients were 0.59 for the Total Difficulties Score and 0.78 for Pro-social Scale.

### Design and analyses

The study utilised a pre–post comparison group design in which stratification by school was adopted to minimise group differences in school and neighbourhood factors and for reasons of feasibility of intervention delivery. With the exception of hypothesis 4, mixed between- and within-participants analyses were employed. Preliminary analyses established that the data did not violate the assumptions required for the analyses planned to test the study hypotheses.

The hypotheses that, relative to the wait-list comparison condition, a reduction in bullying behaviour and victimisation (hypothesis 1a) and an increase in trait EI (hypothesis 2a) would be found for the EL intervention condition, and that the hypothesised change in each case would be greater for those children whose EL scores were initially low (hypothesis 1b and 2b), were tested using three mixed analysis of variances (ANOVAs) with the peer-rated (Guess Who) bullying behaviour and victimisation scores, and the trait emotional intelligence (*TEIQue-CF*) score as the dependent variables, Group (intervention or wait-list comparison) and EL (above or below the median on *ELA-PF*) as the between participants variables, and time (pre-intervention and post-intervention) as the within participants variable.

The hypothesis that, relative to the wait-list comparison condition, an improvement in adjustment would be found for the EL intervention condition (hypothesis 3a) and that the improvement would be greater for those children whose EL scores were initially low (hypothesis 3b), were tested using a mixed multivariate analysis of variance with the Total Difficulties and Pro-Social scores of the Strengths and Difficulties Questionnaire as the dependent variables, Group (intervention or wait-list comparison) and EL (above or below the median on *ELA-PF*) as the between participants variables, and time (pre-intervention and post-intervention) as the within participants variable. A correlational design was employed to test hypotheses 4. The predictions that reductions in bullying and victimisation scores on the Guess Who measure and improvements in adjustment, as assessed by the Total Difficulties and Pro-Social scores of the Strengths and Difficulties Questionnaire would be associated with increases in emotional intelligence (*TEIQue-CF*) and EL (*ELA-PF*), particularly among children who had received the EL intervention, were tested using Pearson's correlation coefficients with the intervention and wait-list comparison group data.

## Results

(1) Bullying behaviours and victimisation will: (a) decrease for participants receiving the EL intervention, relative to those in the wait-list comparison group and (b) decrease more for intervention group participants initially scoring low on EL than for those initially scoring high on EL.

The results of the data analyses are provided in Table 1, from which it can be seen that there was a significant three-way interaction effect between time, group (intervention or comparison) and EL (high or low). As such, main effects were not interpretable and simple effects (pre-post effects for each group based on EL category) were examined. Simple effect analyses for the Low EL group showed a statistically significant effect of the intervention on peer nominations of bullying: interaction between time and group *F*(1, 20) = 5.18, *p* = .03, η_*p*_^2^ = .21. The effect size associated with this interaction is large, partial eta squared of .01 representing a small effect, .06 a moderate effect and .14 a large effect (Cohen, 1988).

**Table 1.** Mixed ANOVA summary table for bullying scores at pre-test and post-test for children of high and low EL in the Intervention or wait-list comparison groups.

Source	df	*F*	*p*	η^2^_*p*_
Time	1	10.23	.003	.20
Group	1	3.49	.07	.78
Time × group	1	1.10	.30	.03
Time × EL	1	0.12	.73	.003
Time × group × EL	1	5.99	.02	.13
Error	41			

**Figure 1.**
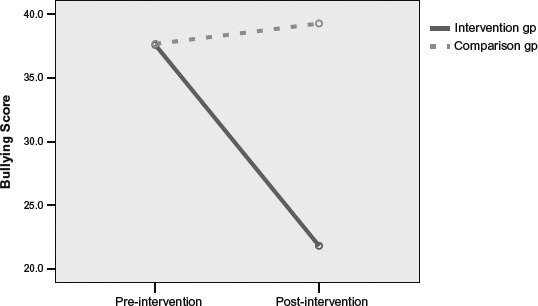
Pre–post intervention differences in peer rated bullying behaviour for children with low emotional literacy in the intervention and comparison groups.

Figure 1 shows the mean differences in bullying scores of children with EL scores (*ELA-PF*), below the median at pre-test. Engagement in bullying behaviour (Guess Who) decreased significantly from pre-test to post-test for those Low EL children who received the intervention, *t*(10) = 2.70, *p* = .02, but for those in the wait-list comparison group it did not significantly decrease, *t*(10)= −0.33, *p* = .75.

**Figure 2.**
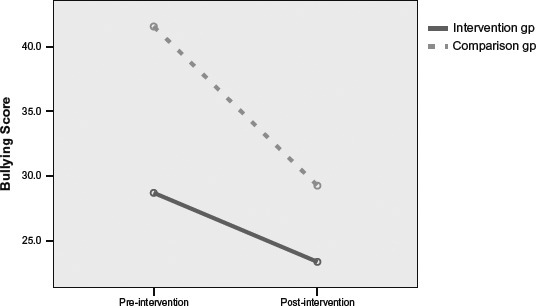
Pre–post intervention differences in peer rated bullying behaviour for children with high emotional literacy in the intervention and comparison groups.

Figure 2 shows the mean differences in bullying scores of children with EL scores above the median. By contrast, the analyses for the High EL group did not show a statistically significant effect of the intervention *F*(1, 21)= 1.17, *p* = .29, η_*p*_^2^ = .05. In this analysis, there was a significant main effect of time, *F*(1, 21) = 7.53, *p* = .012, η_*p*_^2^ = .26 with scores tending to decrease, but not of group, *F*(1, 21) = 2.20, *p* = .15, η_*p*_^2^ = .10.

Table 2 shows the pre-intervention and post-intervention means and standard deviations of Victimisation scores (Guess Who) for children in the intervention and comparison group, scoring high and low on EL. Analyses of these scores indicated a significant main effect of time, *F*(1, 41)= 13.69, *p* = .001, η_*p*_^2^ = .25, but not of group, *F*(1, 41) = 1.57, *p* = .22, η^2^_*p*_ = .04, or EL category, *F*(1, 41) = 1.03, *p* = .32, η^2^_*p*_ = .02 Neither the three-way interaction, nor any of the two-way interactions were significant. This result indicates that victimisation decreased over time irrespective of the child's initial EI score or their receipt of the intervention. The hypothesis of an intervention effect on victimisation was not supported.

**Table 2.** Means and standard deviations of scores on victimisation and adjustment measures at pre-test and post-test for intervention and wait-list comparison group children in the high or low EL categories.

		Intervention group				Comparison group			
	Low EL (*n* = 11)		High EL (*n* = 11)		Low EL (*n* = 11)		High EL (*n* = 12)	
		*M*	SD	*M*	SD	*M*	SD	*M*	SD
Victimisation rating	Pre	16.24	(13.32)	9.92	(8.82)	19.04	(10.84)	17.03	(10.32)
	Post	10.16	(9.01)	8.48	(6.27)	11.76	(10.84)	10.70	(11.67)
Trait emotional intelligence	Pre	3.04	(0.28)	3.58	(0.36)	3.12	(0.28)	3.48	(0.43)
	Post	2.91	(0.39)	3.56	(0.46)	3.94	(0.35)	3.30	(0.36)
Adjustment – SDQ total difficulties	Pre	23.91	(4.87)	17.00	(4.71)	21.91	(5.03)	18.33	(4.64)
	Post	22.89	(4.99)	18.10	(6.71)	19.56	(6.95)	19.36	(5.97)
Adjustment – SDQ pro-social behaviour	Pre	6.18	(2.56)	7.73	(1.19)	4.55	(2.59)	6.83	(3.30)
	Post	6.33	(2.12)	6.60	(1.71)	5.67	(3.12)	6.55	(2.91)

(2) Trait EI will: (a) increase for participants receiving the EL intervention, relative to those in the wait-list comparison group and (b) increase more for intervention group participants initially scoring low on EL, than for those initially scoring high on EL

Table 2 shows the pre-intervention and post-intervention means and standard deviations of trait emotional intelligence (*TEIQue-CF*) scores for children in the intervention and comparison group, scoring high and low on EL. In the analysis of these scores, there was a significant main effect of EL category, *F*(1, 34) = 25.00, *p* = .000, η^2^_*p*_ = .42, but not intervention group or time, *F*(1, 34) = 0.42, *p* = .52, η^2^_*p*_ = .01, or time, *F*(1, 34) = 2.86, *p* = .10, η^2^_*p*_ = .08. This result shows that children in the high EL category consistently score higher on the TEIQue-CF than children in the low EL category. However, the hypothesis of an intervention effect on emotional intelligence was not supported.

(3) Indices of adjustment will: (a) improve for participants receiving the EL intervention, relative to those in the wait-list comparison group and (b) improve more for intervention group participants initially scoring low on EL, than for those initially scoring high on EL.

In the Adjustment analyses, a doubly multivariate analysis was conducted to assess whether there was a difference in the amount of change on the Total Difficulties score and the Pro-Social score of the Strengths and Difficulties Questionnaire between the intervention and wait-list comparison groups, and between the children who scored high and low on EL at pre-test. A significant multivariate effect was found for the main effect of EL category, *F*(2, 34) = 5.48, *p* = .009, η^2^_*p*_ = .24, but not group, *F*(2, 34) = 0.97, *p* = .39, η^2^_*p*_ = .06, or time, *F*(2, 34) = 0.24, *p* = .79, η^2^_*p*_ = .01. Neither the three-way interaction, nor any of the two-way interactions were significant. This finding indicates that the high and low EL groups differ on the linear combination of the two adjustment variables.

Follow-up ANOVAs revealed a significant difference on the Total Difficulties score, *F*(1, 35) = 8.32, *p* = .007, η^2^_*p*_ = .19, but the difference on pro-social behaviour did not reach the *p* < .05 criterion for statistical significance, *F*(1, 35) = 3.35, *p* = .076, η^2^_*p*_ = .09. The pre-intervention and post-intervention means and standard deviations of the adjustment scores (Total Difficulties and Pro-social scale scores on the SDQ) for children in the intervention and comparison group, scoring high and low on EL can be found in Table 2. The ANOVA results indicate that children scoring in the low EL category experienced a higher level of adjustment related difficulties than those scoring in the high EL category, across time and intervention groups. The hypothesis of an intervention effect on the adjustment variables was not supported.

Hypothesis 4 Reductions in bullying and victimisation and improvements in adjustment will be associated with increases in trait EI and EL scores, particularly in the intervention group.

In both the intervention and wait-list comparison group changes over time in the EL, trait EI, and pro-social behaviour measures were highly correlated (see Table 3). However it was only in the intervention group that increases in EL and trait EI were associated with decreases in children's self-reported adjustment difficulties. Likewise, it was only in the intervention group that increases in trait EI were associated with decreases in peer-reported engagement in bullying behaviour. Moderate, but non-significant, decreases in victimisation were associated with increases in EL and trait EI in the intervention group. By contrast, in the comparison group, increases in EL and trait EI scores tended to be associated with increases in victimisation, a statistically significant increase being found between victimisation and trait EI. It can be concluded that increases in emotional intelligence are associated with decreases in bullying behaviour and adjustment difficulties among children who received the EL intervention, but not those in the wait-list comparison group.

**Table 3.** Correlations between pre–post intervention change scores on emotional intelligence, adjustment, bullyin g and victimisation for intervention and comparison group participants.

Pre–post intervention change scores	EL	TEIQue	Bullying	Victim	Total diffs	Pro-social
EL	–	.60**	−.37	−.26	−.51*	.53*
Trait emotional intelligence (TEIQue)	.51*	–	−.52*	−.35	−.56*	.50*
Bullying ratings	.29	.13	–	.23	.15	−.39
Victimisation ratings	.31	.54*	.22	–	.12	−.15
Adjustment – total difficulties	−33	.001	−.11	−.14	–	−.29
Adjustment – pro-social behaviour	72**	.70**	.14	.32	−.12	–

Note: Correlations above the diagonal are for the intervention group and below the diagonal are for the comparison group.

* *p* < .05, **p < 0.01.

## Discussion

This study was designed to evaluate the effects of an EL intervention for peer-identified pupils who bully in four schools where a universal social and emotional learning programme was already in place. A pre–post comparison group design with stratification by school was employed. Hypothesised effects of the intervention and interactions with initial levels of EL were tested for peer-reported engagement in bullying behaviour and levels of victimisation and for self-reported trait EI and adjustment measures. Hypothesised associations between changes in these variables were also investigated.

### Key findings

The intervention was found to have a differential effect on pupils who bully, depending on their initial level of EL. Pupils whose EL score was low at the start of the intervention only showed significant reductions in bullying behaviour if they received the targeted EI intervention. Pupils whose EL score was high at the start of the intervention showed an overall decrease in bullying behaviour whether they were in the intervention or comparison group. While the effect size of these significant reductions in bullying behaviour was large, in contrast to the small effect sizes reported by meta-analyses in the field (Merrell et al., 2008; Ttofi & Farrington, 2011), this is not surprising as effects in this study were assessed only for the children high on bullying behaviour at the outset who were identified for the intervention, not for all the children in the school. One interpretation of the pattern of findings obtained is that children with high EI were able to take advantage of the universal programmes being offered by the school and did not benefit further from a more intensive EI-focused intervention. By contrast children in the low EI group may have required the intervention offered by the more intensive and focused small group EL programme which was targeted on bullying.

This finding has a number of parallels in the EI literature with secondary aged pupils. Qualter et al. (2007) found a positive response on measures such as lateness to class by pupils with low EI, but not high EI, to a universal EL programme delivered in the first year of secondary school. In a different aspect of school life, Petrides et al. (2004) found that among pupils at risk of school drop out, those with low trait EI appeared particularly vulnerable. Hence among pupils with high cognitive abilities, there was little difference in academic attainment between those with high EI and those with low EI. However, among pupils with low cognitive abilities, those with high EI did significantly better academically than those with low EI.

The interaction effect found for bullying was specific to this variable and was not apparent on any of the other outcome measures. The specificity of the effect is somewhat surprising given the moderate associations reported in previous studies between these variables, for example between trait EI and peer-reported aggression (Petrides et al., 2006), between peer reported bullying and the measures of adjustment used in this study (Viding et al., 2009). However, it does at least suggest that the effect is unlikely to be due to factors such as greater tractability per se of problems experienced by pupils in the low EL category. Main effects of EL category were found, with pupils in high the EL category scoring higher on trait EI and lower on adjustment difficulties. These again are consistent with previous findings in the literature (Mavroveli et al., 2009; Qualter et al., 2007; Williams et al., 2009).

When associations between change scores were examined, they were found in a number of cases to be of similar magnitude in both the intervention and the comparison group. It is likely that these associations between change scores on trait EI, EL and pro-social behaviour, primarily reflect their overlapping content domains. By contrast, the finding of significant associations in the intervention group only between decreases in adjustment problems and increases in trait EI or EL cannot be explained by the content or nature of the measures. However, as these measures all rely on self-report, there is the possibility that associated changes reflect factors such as increased positivity relating to the additional small group attention received through participation in the intervention. Such explanations cannot account for the finding of a significant association, in the intervention group only, between increases in self-rated trait EI and reductions in peer-rated bullying behaviour. While it is not possible to conclude that these changes are attributable to the EL content of the intervention, it does suggest that further research is warranted with larger samples to allow change processes models to be tested.

One puzzling result from the correlational analysis was the significant association found between increases in peer rated victimisation and trait emotional intelligence in the wait-list comparison group, particularly as the moderate, non-significant correlations between these variables in the intervention group were negative. It is possible that different processes are operating in the two groups. In the intervention group increases in emotional intelligence may have some protective function, whereas in the comparison group the experience of being victimised may stimulate the developing of coping strategies that are reflected in increased emotional intelligence scores. Again, further research with larger samples would be needed to test such hypothesised process models.

### Limitations and future directions

A number of limitations of the study must be acknowledged. Given the nested nature of the design, of children within school-based groups, use of a multi-level analysis would have been desirable. This was precluded as only four schools were involved, so there were insufficient level 2 units (Snijders & Bosker, 1999). As the sample was stratified by school, direct effects of school might reasonably be assumed to be equivalent across both intervention and comparison conditions. However, possible school by intervention interactions could not be investigated. Following on from the encouraging results of this small scale pilot study, further research using the larger samples required by a multi-level strategy would be necessary to adequately take account of the nested nature of the data.

In the between-groups design employed, there was sufficient power to detect moderate to large effects, and these are likely to be of most relevance to practice. However, the low sample size means that some potentially interesting, albeit small, effects may have been missed. The wait-list comparison group provided a control for the passage of time, but there was no control for the additional adult attention or the focus on bullying received by the intervention group. It is possible that children in the low EL category responded differentially to incidental aspects such as these, rather than to the content of the EL programme. It should also be acknowledge that, despite the efforts of school staff and researchers to frame engagement with the intervention positively, the particular focus on bullying with a subgroup of pupils carries a risk of stereotyping and associated counterproductive effects.

There were limitations also relating to the measures used. In a number of cases, the reliability coefficients were relatively low, namely the EL questionnaire and the total difficulties score of the measure of adjustment used. This may have resulted in attenuated correlations involving these variables and limited the detection of other hypothesised effects. The use of these self-report measures, given the age of the children involved, should be considered in future research. Assessment of adjustment in the study was limited to self report and some children may have selected socially desirable responses, rather than reflecting on their own behaviours. Given the modest correlations often found between child self-report and teacher reports, even on parallel measures, multi-method assessment is commonly recommended (Stanger & Lewis, 1993; Wright & Torrey, 2001). Due to the timing of the intervention in the present study schools were unwilling to commit class teachers to completing the teacher version of the Strengths and Difficulties Questionnaire, prior to and following the intervention period. The possible effects of common method variance in inflating the relationships between intervention group change scores on trait EI and the adjustment measures must be considered. It is worth noting that these correlations are similar in size to the correlation between self-reported trait EI and peer-reported bullying behaviour. Nonetheless, future research should seek to validate these findings using multi-method assessment approaches.

A further set of limitations relate to the variables included in the analyses. Although the focus in this study was on EI, there are clearly many other factors whose role in bullying behaviour in schools have already been demonstrated (Hong & Espelage, 2012). In particular, important socio-cultural variables, such as gender, race, ethnicity and socio-economic status, while examined for the purposes of group matching, could not be included as independent variables in the analysis of this small sample. This is problematical in the light of the established significance in bullying research of gender (Card et al., 2008), minority race and ethnicity status (Vervoort, Scholte, & Oberbeek, 2010) and socio-economic status (Due et al., 2009).

In relation to socio-economic status, it should be noted that the groups in this study were not successfully matched on socio-economic status as the intervention group did not contain any children who were eligible for free school meals, and it is not clear therefore whether the findings would apply to more economically disadvantaged groups of children. Future research should investigate the extent to which these variables moderate the relationship between bullying and EI. However, a comprehensive application of ecological systems theory to school bullying, (Hong & Espelage, 2012) moves beyond the investigation of such associations, identifying a more significant role for structural inequality which may impact also at other levels. For example, the resourcing of schools in particular neighbourhoods is identified as a salient exosystem variable, while at the macrosystem level, school norms and culture may play a role in perpetuating inequality in relation to race, ethnicity, gender and socio-economic background. There is clearly a need in future research on the broader applicability of the findings to investigate associations with socio-cultural factors at a number of levels.

### Implications for educators

The results caution against the adoption of a ‘one size fits all’ approach to targeted bullying interventions in schools. By contrast, in selecting children for interventions designed to develop knowledge and skills negatively associated with bullying behaviour, it is important to establish at the outset that the children have assessed deficits in these areas. It does not appear that children already performing at an average level stand to benefit from participation. The results of this study support the proposal by Brackett, Mayer, and Warner (2004) from a study of ability EI with college students that there may be some threshold level of EI needed for appropriate decision-making in social situations, above which further increases do not confer any further advantage.

This study provides evidence that for some children who bully, those with low EL, a targeted EL intervention can lead to a significant decrease in peer-reported engagement in bullying behaviour. A positive feature of the intervention was that it was delivered by *in situ* school personnel as is widely recommended (Braswell et al., 1997). However caution must be exercised in generalising the results of the study to different school contexts. In all of the schools that participated a national universal social and emotional learning programme was already being implemented and the school personnel involved were experienced in working with small groups of children, albeit primarily on academic tasks. If the intervention was introduced in a school lacking a universal social and emotional learning programme which includes a component on bullying the same pattern of findings may well not be obtained. The focus in this study on individual level variables and targeted interventions is intended to complement the prevailing emphasis in ecological systems approaches on the implementation of universal programmes (Pepler et al., 2004; Swearer & Espelage, 2004), but not to replace them. It does appear that for some children such targeted interventions are necessary, and can be successful provided they are indeed well targeted.
